# Giant Urinary Bladder and Bilateral Giant Hydronephrosis due to Bladder Neck Obstruction: One Case Report and Literature Review

**DOI:** 10.1155/2012/817519

**Published:** 2012-03-07

**Authors:** Mohammed Fadl Tazi, Omar Riyach, Youness Ahallal, Soufiane Mellas, Abdelhak Khallouk, Mohammed Jamal El Fassi, Moulay Hassan Farih

**Affiliations:** ^1^Department of Urology, University Hospital Center Hassan II, Fes, Morocco; ^2^Faculté de Médecine et de Pharmacie de Fès, BP 1893, Km 2.200, Route de Sidi Harazem, Fes, Morocco; ^3^Department of Anatomy, University of Medicine, Fes, Morocco

## Abstract

Bilateral hydronephrosis secondary to urinary obstruction leads to a buildup of back pressure in the urinary tract and may lead to impairment of renal function. Cases of giant hydronephrosis are rare and usually contain no more than 1-2 litres of fluid in the collecting system. Here, we report a rarely seen case with giant urinary bladder and bilateral giant hydronephrosis due to bladder neck obstruction which contains 4000 mL fluid in the collecting system of the kidney mimicking an ascites in an adult male.

## 1. Introduction

Although hydronephrotic kidney is a frequently presenting clinical condition, giant Hydronephrosis is an uncommon entity in adult. The definition of Giant hydronephrosis has been given as the adult renal pelvis containing one liter of urine or 1.6% of body weight. The condition is usually secondary to ureteropelvic junction obstruction, stone diseases, trauma, renal ectopia, and ureterovesical junction obstruction. Hydronephrosis may present an intraabdominal mass with renal swelling features. However, as a result of widespread use of ultrasonography, most cases of hydronephrosis are now diagnosed before the kidney provides any clinical features signs. Massive distension of the kidney may occur and create diagnostic confusion. We report one case of a patient who presented with tense ascites associated with acute renal failure, who was found to have bilateral hydronephrosis. Resolution of hydronephrosis was seen following multiple paracenteses and was accompanied by improvement in renal function.

## 2. Case Report

A 42-year-old male patient was admitted with an 18-month history of progressive abdominal distension and mild dullaching diffuse abdominal pain. He used to have vague abdominal discomfort and did not have specific characteristic pain. Patient did not have any history of fever, jaundice, or vomiting. Also there was no history of weight loss, and he had maintained a good appetite. Hematemesis and melena were also absent. There were no specific urinary or bowel complains.

Clinical examination showed a huge and symmetric distension. No abdominal mass was palpable. The swelling was dull on percussion, but there was no shifting dullness. Examination of other systems was unremarkable.

The serum analysis and complete blood count was performed, and blood urea was 1,65 g/L, creatinine was 78 mg/L, hemoglobin was 11 g/dL, and hematocrit was 51.4%. Blood sugar, serum amylase, liver function tests, and serum calcium, phosphate, and electrolytes were within normal limits. Urine examination did not reveal any abnormality, and urine culture was sterile.

**Figure 1:**
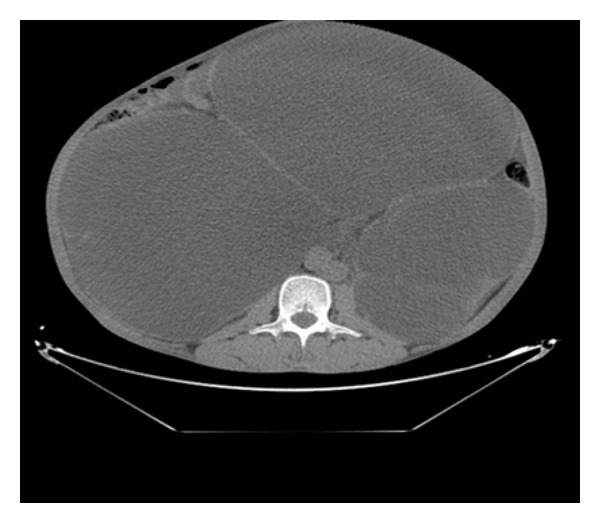
Abdominal CT scan showing massively enlarged bilateral kidney with pressure effect over the bowels.

**Figure 2:**
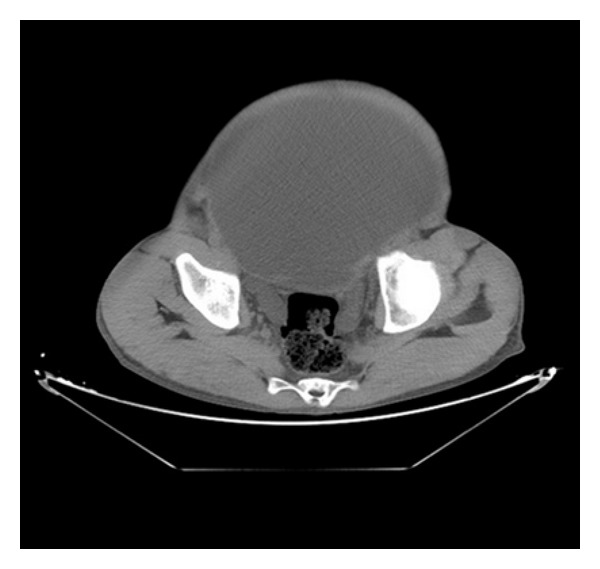
Caudal pelvic CT scan showing a huge urinary bladder.

Diagnostic aspiration from abdominal swelling revealed urine. Ultrasonography revealed a large hypoechoic mass lesion occupying nearly the whole abdomen with multiple septate internal echoes. kidneys were not visualized separately. The liver was displaced upwards. A CT scan of the abdomen, without intravenous contrast, was done to further delineate the site of obstruction. It revealed a giant bilateral hydronephrosis and a huge bladder which occupied the intraperitoneal and pelvic cavities (Figures 1 and 2). No abdominal mass or calculi were seen. Following this, a total of about 20 L of urines fluid was drained by urethral catheterization over a period of 3 days. There was minimal improvement in the renal function, urea was 1,30 g/L, creatinine was 60 mg/L.

Ultrasonography control showed significant regression of the dilatation, but the large bilateral kidney hydronephrosis was still present.

**Figure 3:**
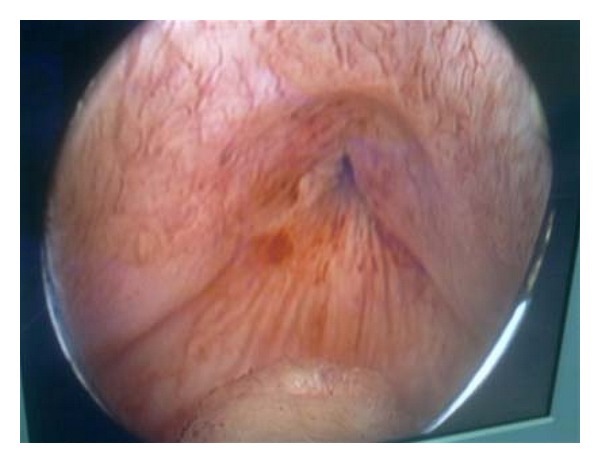
Cystoscopy showing bladder neck obstruction.

The cystoscopy confirmed the huge bladder and showed a bladder neck obstruction (Figure 3), and then a trans-urethral neck incision was performed. Hyporeactive bladder was found in urodynamic analysis, and a cystofix suprapubic bladder catheter was introduced percutaneously. The postoperative period was uneventful. Follow-up US examinations demonstrated nearly complete disappearance of the hydronephrosis; laboratory findings and urine culture were normal.

## 3. Discussion

Giant hydronephrosis is a rare urological entity in patients of all ages [1, 2]. Although several giant hydronephrosis cases have been reported in the English literature, only a few of them contain more than 2 liters of fluid [3, 4]. Chiang et al. reported 4 cases of GH containing 1900 mL, 3400 mL, 2100 mL, and 3200 mL [3]. Yapanoglu et al. 2007 reported a case with 5000 mL of fluid in the collecting system [5]. Schrader et al. reported GH with a kidney of more than 15 kg [6]. Yilmaz and Guney reported hydronephrosis in a 12-year-old boy with 13.5 litres of urine in the collecting system [1]. As of our case, the hydronephrotic kidney contained 20 litres of urine.

The most common cause of GH is ureteropelvic junction obstruction, although stone disease, trauma, renal ectopy, and ureteral tumor have also been reported [2, 7]. In our case, a primary bladder neck obstruction (PBNO) was the cause in which the bladder neck fails to open adequately during voiding, resulting in increased striated sphincter activity or obstruction of urinary flow in the absence of another anatomic obstruction, such as that caused by benign prostatic enlargement in men or genitourinary prolapse in women. PBNO was first described in men by Marion in 1933. Later, in 1973, Turner-Warwick and colleagues advocated the use of urodynamics and voiding cystourethrography to diagnose bladder neck dysfunction in men aged 50 years or younger with a long history of lower urinary tract symptoms (LUTSs).

PBNO can present with a variety of symptoms, including voiding symptoms (decreased force of stream, hesitancy, intermittent stream, incomplete emptying), storage symptoms (frequency, urgency, urge incontinence, nocturia), or a combination of both. Its diagnosis is videourodynamic; the hallmark of which is relative high-pressure, low-flow voiding with radiographic evidence of obstruction at the bladder neck with relaxation of the striated sphincter and no evidence of distal obstruction.

Giant hydronephrosis may present with vague symptoms such as nausea, fatigue or dyspepsia, urinary tract infection, renal insufficiency, or gross hematuria after trauma in adults [8]. However, patients usually remain asymptomatic until late stages, because this situation usually progresses slowly [2]. A giant hydronephrosis seldom fill the entire abdomen as in our patient, and differentiation of the condition from ascites may then be difficult on clinical examination alone. Occasionally, a band of resonance may be present in the opposite flank, but it was not demonstrable in the present case. Some authors noted that a correct preoperative diagnosis of giant hydronephrosis has been made in only 46% of cases. The initial clinical diagnosis of the present case was massive ascites.

Even though diagnostic instruments such as excretory, antegrade, or retrograde urographies, ultrasonography, and CT scans have facilitated the diagnosis of hydronephrosis in the last decades, accurate diagnosis of giant hydronephrosis in individual cases remains challenging [1, 9]. However the first radiological method in GH diagnostics is abdominal ultrasonography, but in many cases differential diagnosis between GH and another cystic formation is difficult. The list of differential diagnosis is wide and includes ovarian cysts, retroperitoneal haematoma, hepatobiliary cysts, mesenteric and hepatobiliary cysts, pseudomyxoma, renal tumour, retroperitoneal tumours, ascites, and splenomegaly [9].

As in our case, giant hydronephrosis due to primary bladder neck obstruction can be treated surgically with unilateral or bilateral transurethral incision of the bladder neck. The main concern with bladder neck incision is the development of postoperative retrograde ejaculation. Retrograde ejaculation is less likely to occur with unilateral incision as opposed to bilateral incision [10, 11].

The essential aim of treatment of GH should be preservation of the kidney. Despite the widespread use of prenatal ultrasound and development of new diagnostic techniques, GH may still be seen in all age groups. In our case the patient underwent unilateral transurethral incision, and the patient was discharged uneventfully at the 15th postoperative day.

## 4. Conclusion

Giant hydronephrosis is a rare clinical entity which may mimic progressive and benign abdominal cystic tumours or massive ascites. Despite the usual use of ultrasonography, hydronephrosis may still be seen in adult population. Contrast-enhanced CT of abdomen and pelvis is the gold standard diagnostic modality for diagnosing giant hydronephrosis and supported with intravenous pyelogram. Giant hydronephrotic kidney can cross the midline and pose as a diagnostic dilemmar; therefore, we emphasise that in an obscure case of ascites, the possibility of hydronephrosis has to be considered before paracentesis is attempted.
